# Long-Term Effects of Aircraft Noise Exposure on Vascular Oxidative Stress, Endothelial Function and Blood Pressure: No Evidence for Adaptation or Tolerance Development

**DOI:** 10.3389/fmolb.2021.814921

**Published:** 2022-01-31

**Authors:** Katie Frenis, Sanela Kalinovic, Benjamin P. Ernst, Miroslava Kvandova, Ahmad Al Zuabi, Marin Kuntic, Matthias Oelze, Paul Stamm, Maria Teresa Bayo Jimenez, Agnieszka Kij, Karin Keppeler, Veronique Klein, Lea Strohm, Henning Ubbens, Steffen Daub, Omar Hahad, Swenja Kröller-Schön, Michael J. Schmeisser, Stefan Chlopicki, Jonas Eckrich, Sebastian Strieth, Andreas Daiber, Sebastian Steven, Thomas Münzel

**Affiliations:** ^1^ Department of Cardiology, Cardiology 1, Laboratory of Molecular Cardiology, University Medical Center of the Johannes Gutenberg University, Mainz, Germany; ^2^ Boston Children’s Hospital and Harvard Medical School, Department of Hematology/Oncology, Boston, MA, United States; ^3^ Department of Otorhinolaryngology, University Medical Center Bonn (UKB), Bonn, Germany; ^4^ Jagiellonian Centre for Experimental Therapeutics (JCET), Jagiellonian University, Krakow, Poland; ^5^ German Center for Cardiovascular Research (DZHK), Partner Site Rhine-Main, Mainz, Germany; ^6^ Institute for Microscopic Anatomy and Neurobiology, University Medical Center of the Johannes Gutenberg-University, Mainz, Germany; ^7^ Focus Program Translational Neurosciences (FTN), University Medical Center of the Johannes Gutenberg-University, Mainz, Germany; ^8^ Department of Pharmacology, Medical College of the Jagiellonian University, Krakow, Poland; ^9^ Center for Thrombosis and Hemostasis, University Medical Center of the Johannes Gutenberg-University, Mainz, Germany

**Keywords:** acute and chronic aircraft noise exposure, hypertension, endothelial dysfunction, oxidative stress, hearing threshold by audiometry, stress adaptation

## Abstract

Transportation noise is recognized as an important cardiovascular risk factor. Key mechanisms are noise-triggered vascular inflammation and oxidative stress with subsequent endothelial dysfunction. Here, we test for adaptation or tolerance mechanisms in mice in response to chronic noise exposure. C57BL/6J mice were exposed to aircraft noise for 0, 4, 7, 14 and 28d at a mean sound pressure level of 72 dB(A) and peak levels of 85 dB(A). Chronic aircraft noise exposure up to 28d caused persistent endothelial dysfunction and elevation of blood pressure. Likewise, reactive oxygen species (ROS) formation as determined by dihydroethidium (DHE) staining and HPLC-based measurement of superoxide formation in the aorta/heart/brain was time-dependently increased by noise. Oxidative burst in the whole blood showed a maximum at 4d or 7d of noise exposure. Increased superoxide formation in the brain was mirrored by a downregulation of neuronal nitric oxide synthase (*Nos3*) and transcription factor *Foxo3* genes, whereas *Vcam1* mRNA, a marker for inflammation was upregulated in all noise exposure groups. Induction of a pronounced hearing loss in the mice was excluded by auditory brainstem response audiometry. Endothelial dysfunction and inflammation were present during the entire 28d of aircraft noise exposure. ROS formation gradually increases with ongoing exposure without significant adaptation or tolerance in mice in response to chronic noise stress at moderate levels. These data further illustrate health side effects of long-term noise exposure and further strengthen a consequent implementation of the WHO noise guidelines in order to prevent the development of noise-related future cardiovascular disease.

**GRAPHICAL ABSTRACT:**
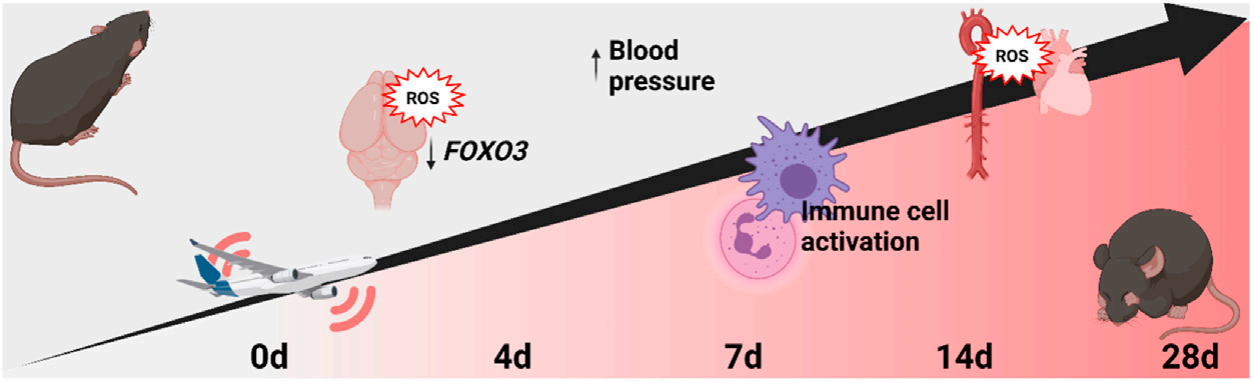


## Introduction

Transportation noise is increasingly recognized as an important cardiovascular risk factor (Munzel et al., 2020; Munzel et al., 2021). Noise research has established that this environmental stressor is associated with cerebro/cardiovascular health side effects such as hypertension (Jarup et al., 2008; Fuks et al., 2017), myocardial infarction (Sorensen et al., 2012; Bai et al., 2020), and stroke (Sorensen et al., 2011; Floud et al., 2013) as also summarized by the recent WHO Environmental Noise Guidelines for the European Region (Kempen et al., 2018). Short-term nighttime aircraft or railway noise exposure in healthy subjects and patients with established coronary artery disease causes endothelial dysfunction, hypertension and increased stress hormone release (Schmidt et al., 2013; Schmidt et al., 2015; Herzog et al., 2019). Further translational studies in a recently developed animal model of aircraft noise confirmed the negative impact of noise on endothelial function, vascular oxidative stress and inflammation and extended these observations to the brain (Munzel et al., 2017; Kroller-Schon et al., 2018).

Noise may exert its effects either directly by causing inner ear damage, or indirectly through the emotional and the cognitive perception of sound (auditory and non-auditory effects) (Basner et al., 2014). Non-auditory effects including disturbance of sleep and communication may lead to emotional reactions such as annoyance, leading to amygdala and hypothalamic-pituitary-adrenal (HPA) axis activation with subsequent increased stress hormone release (Daiber et al., 2019; Osborne et al., 2020). Noise has also a negative impact on the cognitive development of children (Stansfeld et al., 2005) such as learning and memory and also may cause mental disease including in particular Alzheimer’s disease (Cantuaria et al., 2021), depression and anxiety disorders (Beutel et al., 2016). Previous studies have demonstrated that annoyance and mental stress in response to noise is strongly connected to an adaptation/habituation processes (Stansfeld, 1992). In controlled settings, however, we recently demonstrated that the experience of a previous nighttime noise sensitized rather than desensitized the vasculature to develop endothelial dysfunction (Schmidt et al., 2013). In addition, other groups established that cardiac arousal responses caused by noise do not display habituation (Griefahn et al., 2008; Basner et al., 2011).

In light of a recent experimental study reporting on habituation/adaptation of mice to loud noise in the United Kingdom underground system, partially due to adaptation of their immune system (Abolins et al., 2017), we sought to determine the cardiovascular/cerebral side effects of long-term low-level aircraft noise exposure (up to 28d), whether these side effects increase over time, or whether there is evidence for adaptation or even tolerance development. In addition, we tested for the first time whether animals develop significant hearing loss in response to the chosen aircraft noise levels.

## Materials and Methods

### Materials

For isometric tension studies, nitroglycerin (GTN) was used from a Nitrolingual infusion solution (1 mg/ml) from G.Pohl-Boskamp (Hohenlockstedt, Germany). L-012 [8-amino-5-chloro-7-phenylpyrido (3,4-d)pyridazine-1,4-(2H,3H)dione sodium salt] was purchased from Wako Pure Chemical Industries (Osaka, Japan). Endothelin-1 was obtained from Bachem AG (Bubendorf, Switzerland). The QuantiTect probe RT-PCR Kit was purchased from Qiagen (Hilden, Germany) and TaqMan probes from Applied Biosystems (Darmstadt, Germany). The Bradford reagent was obtained from BioRad, Munich, Germany. All other chemicals were of analytical grade and were obtained from Sigma-Aldrich, Fluka or Merck.

### Animals and Treatment

All animals were treated in accordance with the Guide for the Care and Use of Laboratory Animals as adopted by the United States National Institutes of Health and approval was granted by the Ethics Committee of the University Hospital Mainz and the Landesuntersuchungsamt Rheinland-Pfalz (Koblenz, Germany; permit number: 23 177-07/G 15-1-094). Male C57BL/6J mice were purchased from Janvier and allowed to acclimatize for 2 weeks prior to any exposure. Noise was applied for 28, 14, 7, or 4 days or mice remained unexposed. Animals were transported to the noise exposure chamber at appropriate time points such that all would be exposed in tandem and sacrificed on the same day. The exact exposure conditions including the details on the MP3 player and decibel meter are explained in detail in references (Munzel et al., 2017; Kroller-Schon et al., 2018; Munzel et al., 2018). As a proof-of-concept, some mice were treated with the angiotensin-converting enzyme (ACE) inhibitor captopril (50 mg/kg/d in the drinking water (Canby et al., 1989) for 7 days) starting 3 days prior to 4 days of noise exposure (number of mice: 8 unexposed controls, 6 noise and 9 noise + captopril). After the indicated duration of noise exposure, animals were killed under isoflurane anesthesia by transection of the diaphragm and removal of the heart, aorta, and other organs.

### Auditory Brainstem Response Audiometry

Evaluation of hearing thresholds was carried out before and after interventions using click-dependent auditory brainstem response (ABR) audiometry as previously described (Arpornchayanon et al., 2011; Arpornchayanon et al., 2013; Land et al., 2016). Auditory testing was carried out in a soundproof chamber (IAC 400-a, Industrial Acoustics Company, Niederkrüchten, Germany). A custom-made speaker as well as a calibrating microphone were placed approximately 2.5 cm from the pinna while the contralateral ear was plugged with soft ear wax (Ohropax, Werheim, Germany). ABRs were derived under usage of a custom-made setup with a multifunction I/O card (National Instruments Corp., Austin, TX, United States) equipped with AudiologyLab 3.8 (Otoconsult, Frankfurt am Main, Germany). Hearing thresholds by registration of ABRs were determined using clicks (100 μs duration) in increments of 3 dBs (128 repetitions per increment). The bandpass filter was set between 200 and 5,000 Hz.

### Non-invasive Blood Pressure Measurements

NIBP measurements were performed on days 0, 4, 7, 14, and 28 of the noise exposure regimen (CODA 2, Kent Scientific, Torrington, United States). Preceding the baseline measurement at day 0, animals were trained for NIBP measurements three times to prevent stress reactions from restraint. Animals entered restraining tubes freely and were then placed on a preheated plate (32°C) and allowed to acclimate for 20 min. Each session of measurements was comprised of 15 sequential blood pressure measurements, the first 5 of which were discarded as acclimatory cycles. Data points thereby represent the mean value of ten measurements per animal on the given treatment day. Feng et al. proofed accuracy of this method compared to radiotelemetric measurement (Feng et al., 2008).

### Isometric Tension Studies

Aortas were dissected from the sacrificed animals and the perivascular fat was removed. A 4 mm ring was sectioned from the thoracic aorta, which were then mounted on force transducers in the organ bath systems. The rings were pre-constricted with prostaglandin F_2α_ to approximately 80% maximal tone (versus the tone induced by KCl bolus) and concentration-relaxation curves in response to increasing concentrations of acetylcholine (ACh) and nitroglycerin (GTN) were performed as described (Munzel et al., 1995; Oelze et al., 2013).

### Determination of Nitrite Concentration in Plasma

The concentrations of nitrite were measured by ENO-20 NOx Analyzer (Eicom Corporation, Tokyo, Japan), based on the liquid chromatography method with post column derivatization with Griess reagent and subsequent UV/Vis detection (Kus et al., 2015; Steven et al., 2020). Prior to the analysis, the plasma samples were precipitated with methanol at the ratio of 1:1 (v/v), mixed and subsequently centrifuged (10,000 x g, 10 min, 4°C). 10 µl of supernatant was injected into the HPLC system. Next, nitrite was separated on a NO-PAK column (4.6 μm × 50 mm; Eicom) and mixed with the Griess reagent in the reaction coil to form a purple diazo compound. The absorbance of nitrite (tR = 4.9 min) was measured at a wavelength of 540 nm.

### Superoxide Anion Detection by Dihydroethidium Staining and HPLC

Vascular reactive oxygen species (ROS) formation was determined using dihydroethidium (DHE, 1 µM)-dependent fluorescence microtopography in aortic cryo-sections as described (Oelze et al., 2006; Wenzel et al., 2008). ROS-derived red fluorescence was detected using a Zeiss Axiovert 40 CFL microscope, Zeiss lenses and Axiocam MRm camera. Oxidative stress and superoxide were also measured by a modified HPLC-based method to quantify 2-hydroxyethidium levels as previously described (Zhao et al., 2005; Wenzel et al., 2008). Tissue of aorta, heart, or frontal cortex was incubated with 50 µM DHE for 30 min at 37°C in PBS buffer. Tissues were snap-frozen and stored at −80°C until they were homogenized (glass/glass) in 50% acetonitrile/50% PBS (brain frontal cortex and heart) or pulverized in a mortar under liquid nitrogen and resuspended in homogenization buffer (aorta), centrifuged and 50 µl of the supernatant were subjected to HPLC analysis. The system consisted of a control unit, two pumps, mixer, detectors, column oven, degasser and an autosampler (AS-2057 plus) from Jasco (Groß-Umstadt, Germany) and a C_18_-Nucleosil 100-3 (125 × 4) column from Macherey and Nagel (Düren, Germany). A high pressure gradient was employed with acetonitrile and 50 mM citrate buffer pH 2.2 as mobile phases with the following percentages of the organic solvent: 0 min, 36%; 7 min, 40%; 8–12 min, 95%; 13 min, 36%. The flow was 1 ml/min and DHE was detected by its absorption at 355 nm whereas 2-hydroxyethidium was detected by fluorescence (Ex. 480 nm/Em 580 nm). Of note, the absolute superoxide concentration per mg of protein levels may also be affected by the procedure of normalization to the absolute amount of different tissues and resulting protein content. In addition, the penetration depth of DHE may be more efficient in “thin” tissues such as aorta than in “thicker” tissue pieces as used for the heart.

### Reactive Oxygen Species Detection by L-012 Chemiluminescence

Oxidative burst was measured in fresh citrate blood upon dilution 1:50 and stimulation with zymosan A (50 μg/ml) as well as phorbol ester dibutyrate (10 µM) in PBS buffer containing Ca^2+^/Mg^2+^ (1 mM) by L-012 (100 µM) enhanced chemiluminescence (ECL) using a Mithras2 chemiluminescence plate reader (Berthold Technologies, Bad Wildbad, Germany) (Daiber et al., 2004; Oelze et al., 2014b). L-012 [8-amino-5-chloro-7-phenylpyrido (3,4-d)pyridazine-1,4-(2H,3H)dione sodium salt] was purchased from Wako Pure Chemical Industries (Osaka, Japan). NOX-2 as the source of ROS in this assay was previously confirmed by absence of oxidative burst in whole blood of noise-exposed Nox2 knockout mice (Kroller-Schon et al., 2018).

### ELISA Measurement

ELISA for soluble Nox2-derived peptide was measured from serum of 1, 2 or 4d noise exposed mice and purchased from 2BEEscientific (E13651327). For performing the ELISA we strictly followed the instructions of the vendor.

### Western Blot and Dot Blot Analysis of Proteins

Tissues were harvested and snap-frozen. Snap-frozen tissues were ground into a powder under liquid nitrogen for protein extraction and quantification. For western blotting, procedures were similar to those described previously (Oelze et al., 2014a; Steven et al., 2017). 25 µg of protein was loaded into the gel for analysis of NOX-2 (gp91^phox^, mouse monoclonal, 1:1,000, BD Biosciences, United States) and endothelin-1 (ET-1, rabbit polyclonal, 1:5,000, Abcam, Cambridge, MA, United States). The gel was run at low voltage for 15 min, then increased to 200 V for 1.5–2 h. Following sufficient running, proteins were transferred onto a Protran BA85 (0.45 µm) nitrocellulose membrane (SchleicherandSchuell, Dassel, Germany) at 250 mA for 2.5 h. Following transfer, the membranes were cut for individual antibody analysis and allowed to incubate at 4° overnight. Following several washing steps with PBS-T, the membranes were incubated for 2 h at room temperature with the appropriate secondary antibody (HRP-conjugated anti-mouse secondary antibody 1 and anti-rabbit secondary antibody 2, both 1:10.000 dilution, Vector Laboratories, Burlingame, CA, United States). Positive bands were detected using ECL development (Thermo Fisher, 32 106, Millipore, ab5605, 1:750) and Chemilux Imager (CsX-1400 M, Intas). Densitometric quantification was performed using ImageJ software.

Dot blot analysis was done using 1 µl of plasma or 30 µg of protein isolated from tissues per slot. The samples were loaded onto a nitrocellulose membrane under vacuum by a Minifold I vacuum Dot-Blot system (SchleicherandSchuell, Dassel, Germany), then dried at 60° for 1 hour to adhere the membranes. Following drying, the dots were visualized with ponceau S solution, which was then removed, the membrane washed, and incubated with primary antibody for malondialdehyde (MDA)-positive proteins (1:1,000, Calbiochem, Darmstadt, Germany) to determine lipid peroxidation and for 3-nitrotyrosine (3-NT)-positive proteins, 1:1,000, Upstate Biotechnology, MA, United States). Next, the membranes were incubated with a peroxidase-coupled secondary antibody (GAM-POX and GAR-POX, 1:10,000) (Vector Laboratories, CA, United States). Procedure proceeds as stated according to western blot protocol.

### Quantitative Reverse Transcription Real-Time PCR

Total mRNA from brain tissue was isolated using the acid guanidinium-thiocyanate-phenol method (Chomczynski and Sacchi, 1987). 125 ng of total RNA was used for quantitative reverse transcription real-time PCR (qRT-PCR) analysis using QuantiTect Probe RT-PCR kit (Qiagen) as described previously (Hausding et al., 2013; Oelze et al., 2014b). Primer-Probe-Mixes purchased from Applied Biosystems, Foster City, CA were used to analyze the mRNA expression patterns of neuronal NO synthase (*nNOS*, Mm01208059_m1), VCAM-1 (*vcam-1*, Mm00449197_m1), and FOXO3 (*foxo-3*, Mm01185722_m1). All samples were normalized on the TATA box binding protein (TBP, Mm_00446973_m1) as an internal control. For quantification of the relative mRNA expression the comparative ΔΔCt method was used. Gene expression of target gene in each sample was expressed as the percentage of wildtype.

### Histological and Immunohistochemical Staining of Aortic Rings

Paraffin-embedded aortic samples were stained with primary antibodies against 3-nitrotyrosine (3-NT) (1:200, Merck-Millipore, Darmstadt, Germany) (Oelze et al., 2014b). ET-1 staining was performed using specific antibodies (Pierce #MA3-005: 1:200; Abcam #117757: 1:450) (Munzel et al., 2017). NOX-2 staining was conducted using a specific antibody (mouse monoclonal, 1:200 dilution, #LS-B12365, LS Bio) (Frenis et al., 2021a). Depending upon the species of primary antibodies, appropriate biotinylated (anti-mouse: Vector Lab., Burlingame, CA; anti-rabbit: Thermo Fisher Scientific, Waltham, MA) secondary antibodies were used at dilutions according to the manufacturer’s instructions. For immunochemical detection ABC reagent (Vector) and then DAB reagent (peroxidase substrate Kit, Vector) were used as substrates. Quantification was performed using Image ProPlus 7.0 software (Media Cybernetics, Rockville, MD).

### Statistical Analysis

Results are expressed as mean ± SEM. For audiometry a two-tailed paired *t*-test was used. For nitrite, DHE-HPLC, DHE-staining, NIBP, rt-PCR mRNA measurements and oxidative burst, one-way ANOVA with Tukey’s multiple comparison test was used. For isometric tension studies and blood pressure, 2-way ANOVA with Tukey’s correction was used. All calculations in Prism for Windows, version 9.01, GraphPad Software Inc. Testing for equal variance and normal distribution was performed using the same software. *p*-values < 0.05 were considered as statistically significant.

## Results

### Effects of Long-Term Aircraft Noise on Blood Pressure and Endothelial Function

**FIGURE 1:**
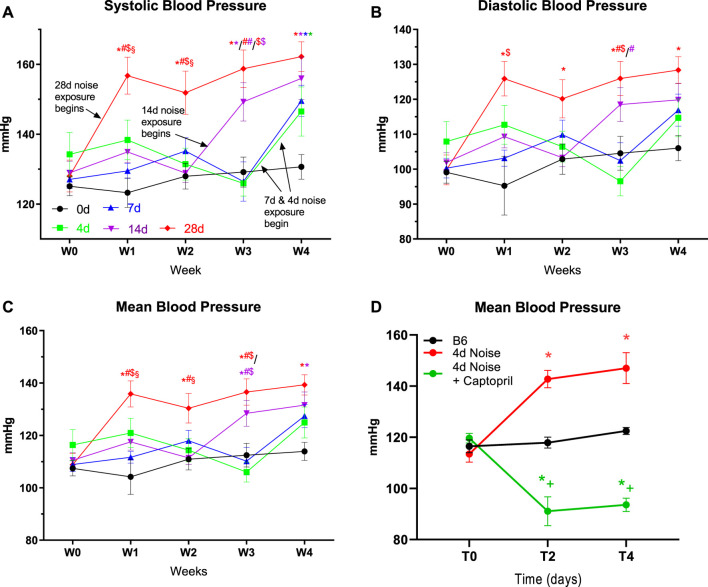
Effects of long-term noise on blood pressure. Blood pressure was evaluated weekly over the course of treatment. **(A, B, C)** Blood pressure increased significantly in every noise-exposed group by the time point following their initial noise exposure in systolic, diastolic, and mean pressures. **(D)** Therapy with the ACE inhibitor captopril prevented blood pressure increase by noise at 2 or 4 days of exposure. Data are mean ± SEM. Statistical analysis with two-way ANOVA with Tukey’s multiple comparison test. Points represent the mean of the group, averaging of 10 measurement cycles per animal, n = 6–9. **(A-C)** *p* < 0.05: * vs respective to 0d, # to 4d, $ to 7d, § to 14d and + to 28d; **(D)** + to noise exposed group. W0-W4 defines the week of noise exposure.

**FIGURE 2:**
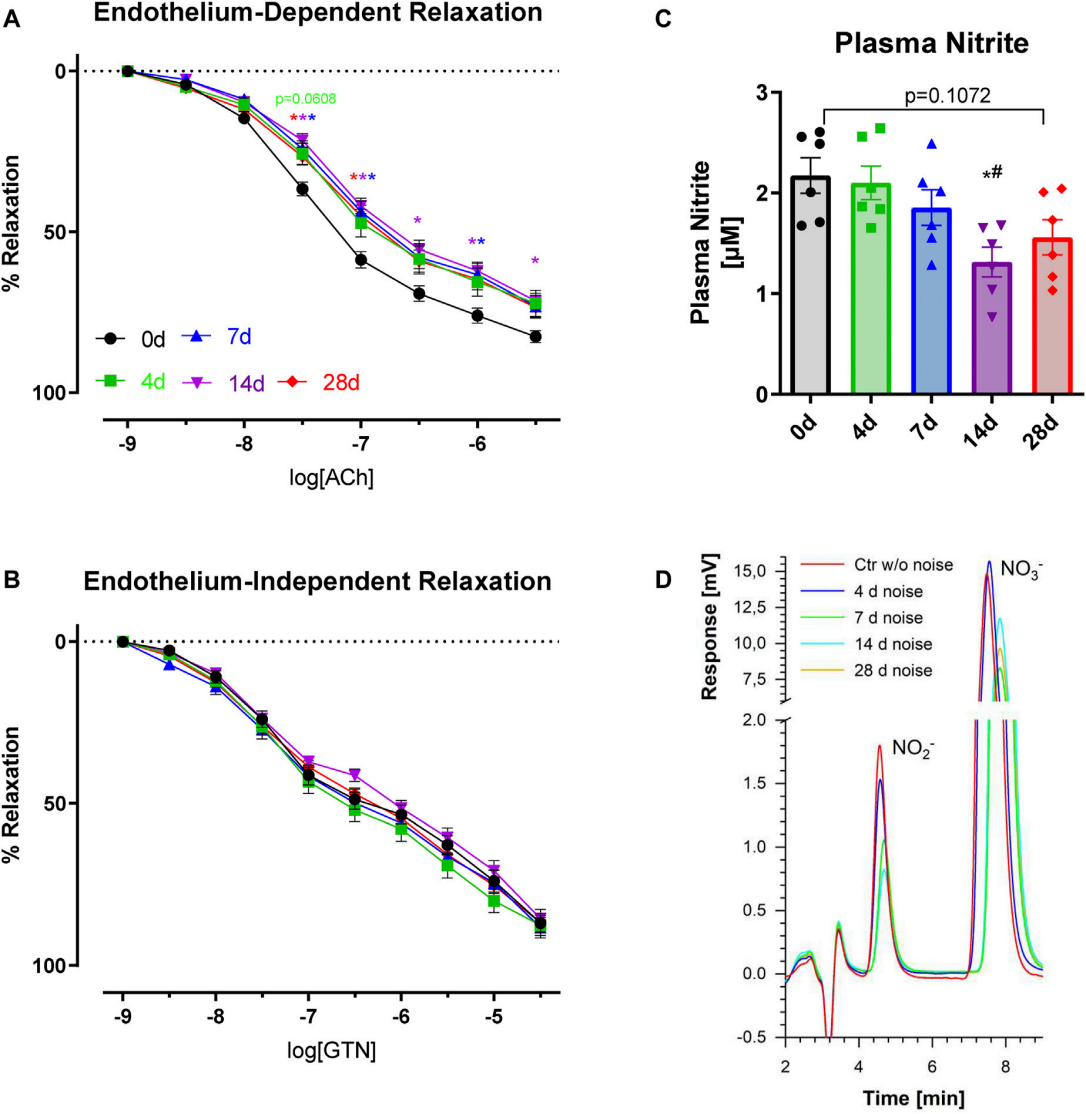
Effects of long-term noise exposure on vascular NO formation and endothelium-dependent vasorelaxation. **(A)** Noise impaired endothelial function in all groups studied to a similar degree. **(B)** Endothelium-independent vasorelaxation to the organic nitrate glyceryl trinitrate (GTN) was not impaired. **(C)** Levels of plasma nitrite were reduced after 14d of noise. **(D)** Representative chromatograms are shown for nitrite determination by HPLC. Data points for **(A,B)** are from individual animals, n = 16–20. Statistical analysis with 2-way ANOVA with Tukey’s correction. Data points for **(C)** are pooled plasma samples, representing two to four animals per point. Statistical analysis with 1-way ANOVA with Tukey’s multiple comparison test. *p* < 0.05: * vs respective to 0d, # to 4d, $ to 7d, § to 14d and + to 28d.

Blood pressure measurements were conducted weekly throughout the noise exposure regimen (Figure 1). At baseline, the mean systolic pressure was 124 mmHg. Blood pressure was measured for all selected animals at every time point, revealing an elevation of blood pressure at the subsequent measurement. This effect was seen in all the noise-exposed groups and was most notable in the 28d group. Systolic pressures rose to a maximum of 178 mmHg and a mean of 153 mmHg over all the exposure groups, indicating a robust but variable hypertensive response to noise. Of note, treatment with the ACE inhibitor captopril completely prevented the blood pressure increase by noise after 4 days of exposure (Figure 1D). In the aorta, there was approximately a ∼15% reduction in endothelium-dependent vasodilative ability irrespective of the exposure time (4d-28d), while no change in endothelium-independent response was noted over the noise exposure time course (Figures 2A,B). NO signaling disruption was highlighted in a gradual reduction in nitrite levels in the plasma, a surrogate parameter for NO bioavailability, indicating that an oxidative phenotype was present, though the progressive decline reached a plateau by 14-28d (Figure 2C).

### Effects of Long-Term Aircraft Noise on Vascular, Cardiac and Circulatory Oxidative Stress

**FIGURE 3:**
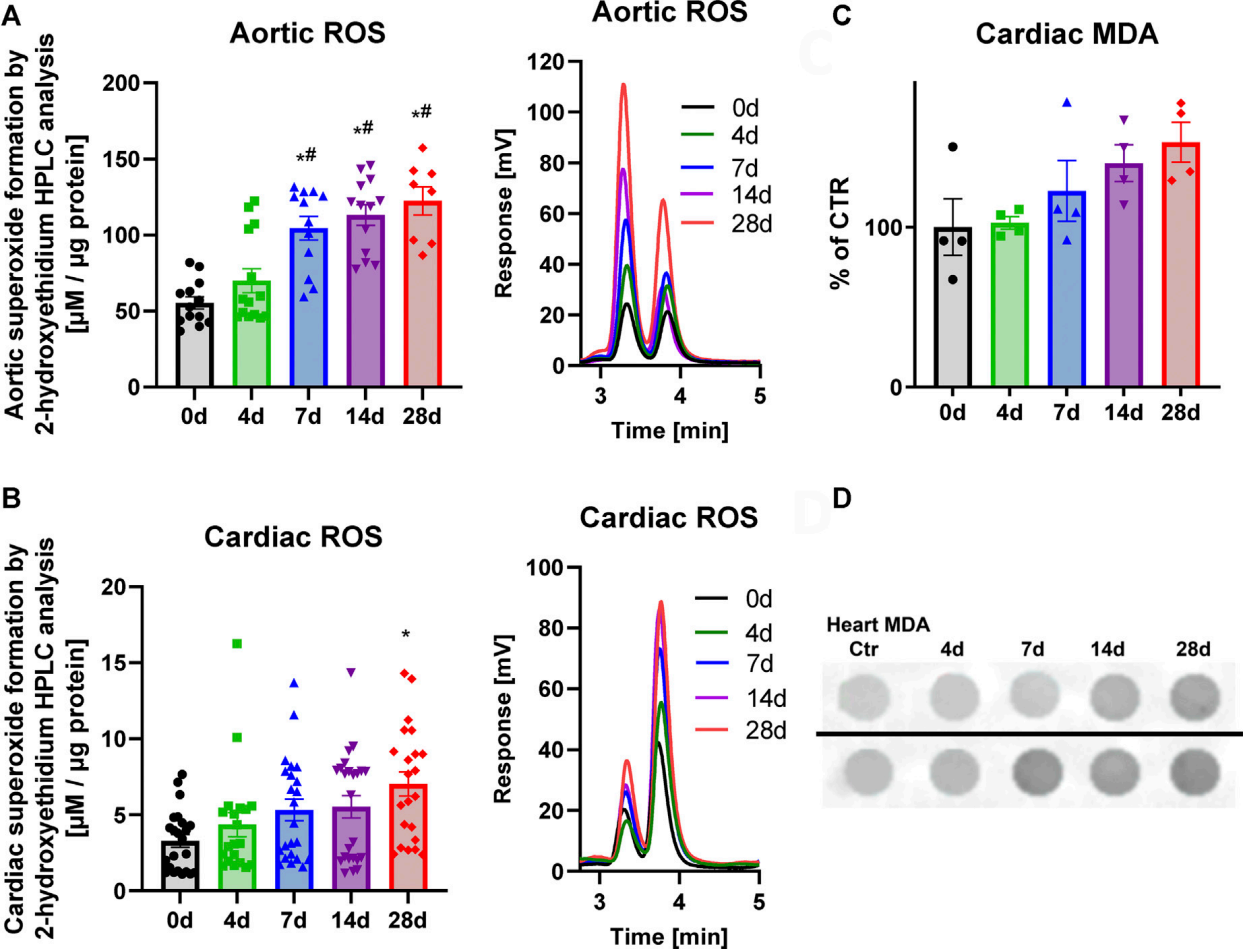
Effects of long-term aircraft noise exposure on vascular and cardiac ROS formation. Levels of superoxide (measured with an HPLC-based dihydroethidium method) showed a progressive increase during noise exposure in both aorta **(A)** and heart **(B)**. Representative chromatograms for measurements are alongside the quantifications. **(C)** Lipid peroxidation was assessed by MDA-positive proteins in the heart and showed a gradual increase with the noise exposure time. **(D)** Representative original dot blot images are shown for all groups. Data points represent individual animals, n = 12–14 **(A)** and n = 22–24 **(B)**. Data points for **(C)** are pooled heart samples, representing two to three animals per point. Statistical analysis was done with one-way ANOVA with Tukey’s multiple comparison test. *p* < 0.05: * vs respective to 0d, # to 4d, $ to 7d, § to 14d and + to 28d.

**FIGURE 4:**
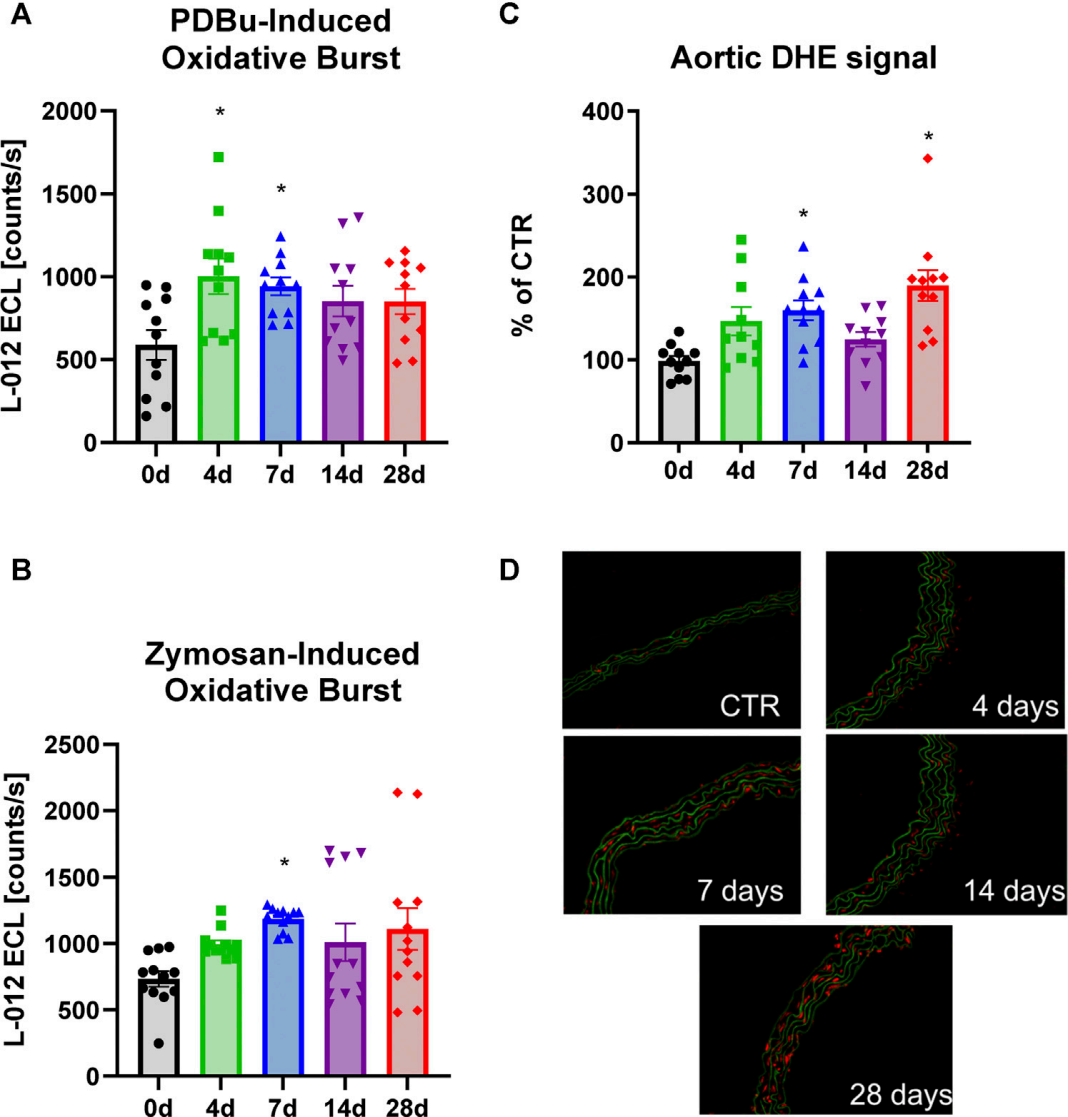
Effects of long-term noise exposure on ROS formation in whole blood and aorta. Stimulation of oxidative burst in whole blood with the phorbol ester (PDBu) **(A)** or the fungal pyrogen (zymosan A) **(B)** indicates that noise induces a heightened ROS response by leukocytes. **(C)** In aorta, staining with DHE revealed a gradual trend for increased ROS levels along with the increasing duration of noise treatment. **(D)** Representative DHE stainings are shown for all groups; red fluorescence indicates ROS formation and green fluorescence come from the autofluorescence of the basal laminae. Data points for **(A,B)** are means from 8 replicates of pooled whole blood from the indicated number of animals per group. Data points for **(C)** represent means of two to four stainings per individual animal. Statistical analysis with one-way ANOVA with Tukey’s multiple comparison test. *p* < 0.05: * vs respective to 0d, # to 4d, $ to 7d, § to 14d and + to 28d.

**FIGURE 5:**
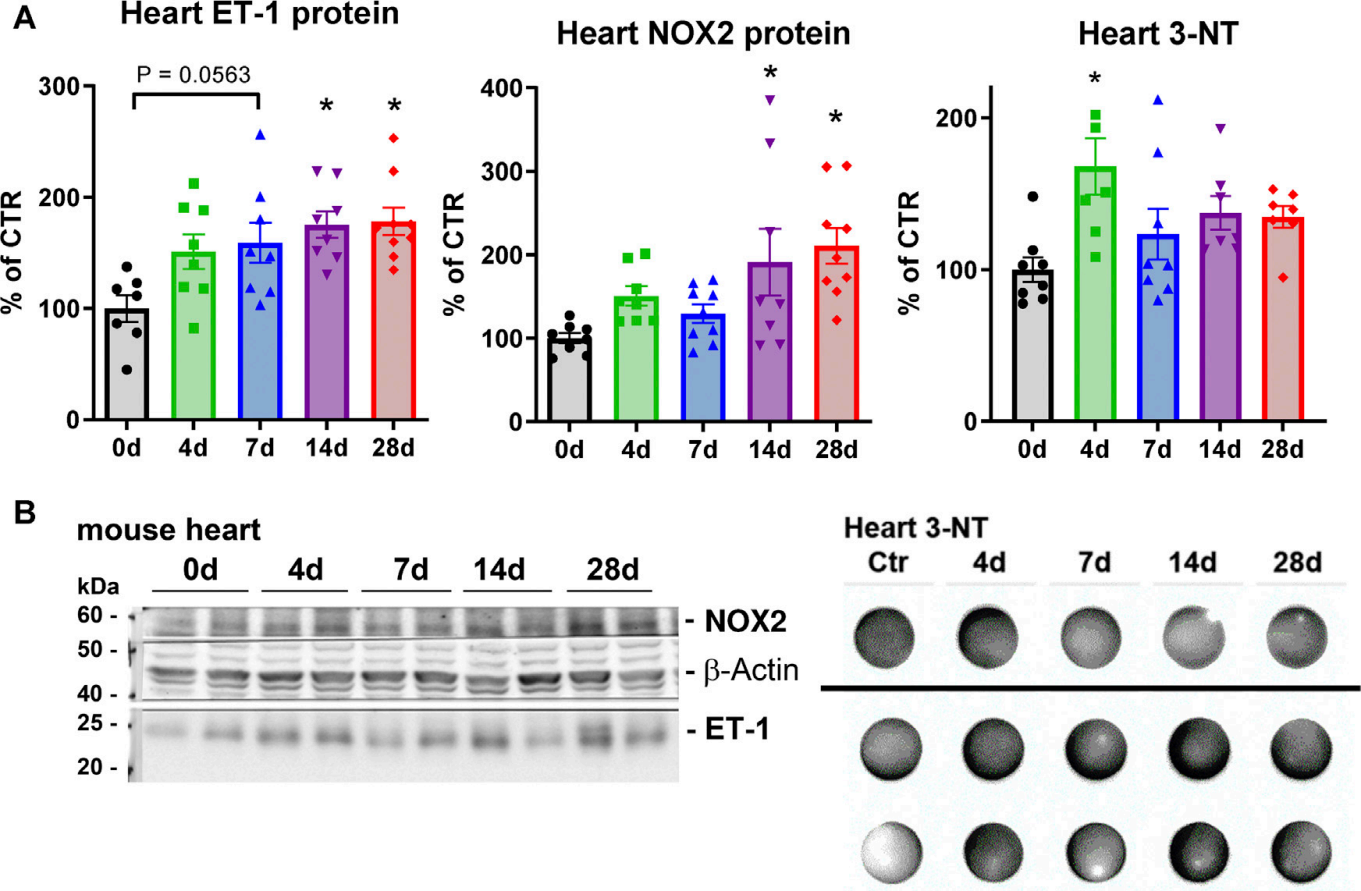
Effects of long-term noise exposure on heart immunoblotting for endothelin-1, NOX2 and 3-NT. **(A)** Cardiac ET-1 and NOX-2 protein expression by Western blotting as well as levels of 3-NT-positive proteins by dot blotting. **(B)** Representative original blotting images for the densitometric quantifications. Data points are pooled heart samples, representing 2 animals per point. Statistical analysis with one-way ANOVA with Tukey’s multiple comparison test. *p* < 0.05: * vs respective to 0d.

**FIGURE 6:**
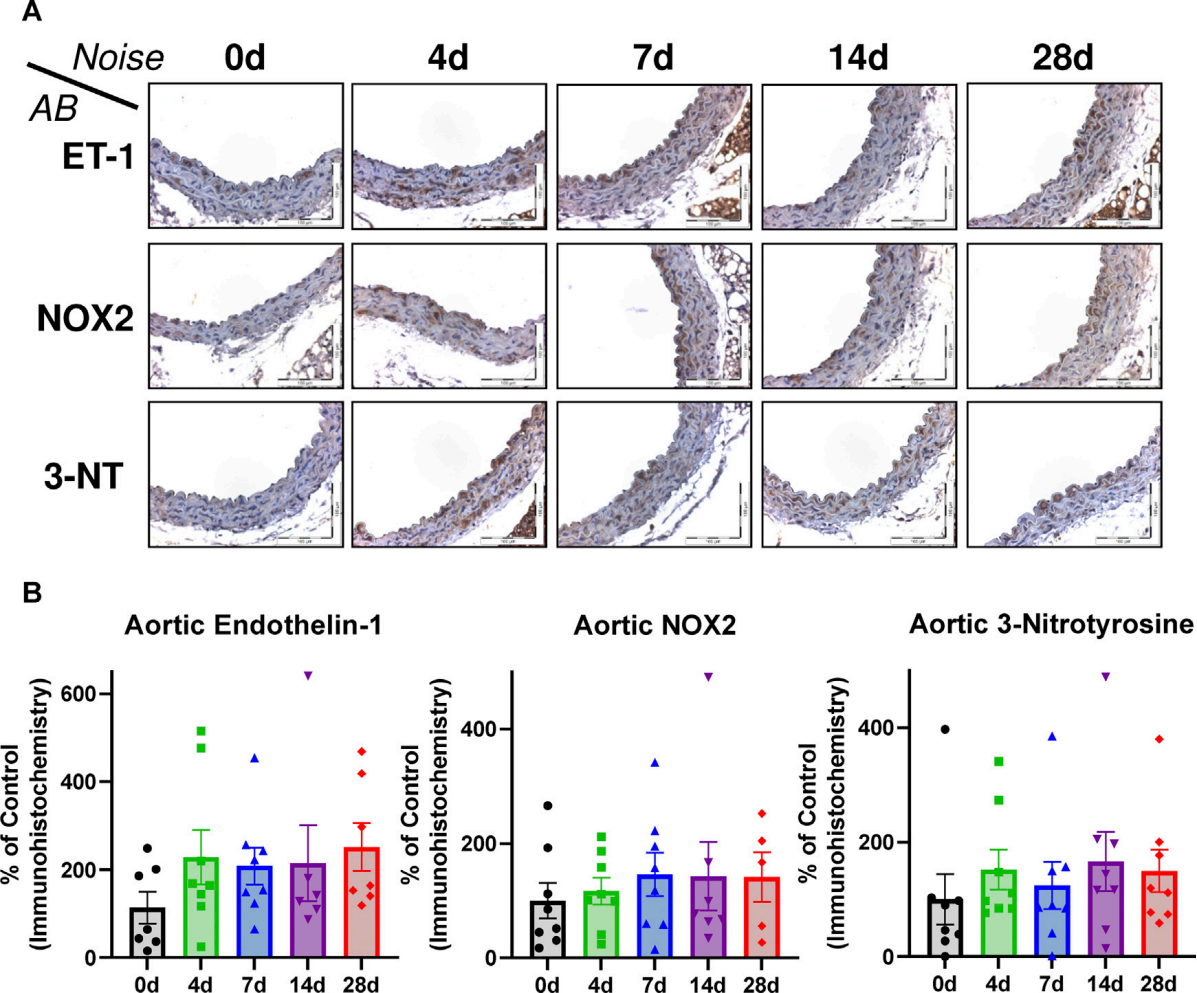
Effects of long-term noise exposure on aortic immunohistochemistry for endothelin-1, NOX2 and 3-NT. **(A)** Representative immunohistochemical stainings of aortic paraffin sections (20x magnification). **(B)** Densitometric quantification of immunohistochemical staining for ET-1, NOX2 and 3-NT in aortic tissue. Data points represent individual animals, n = 5–8. Statistical analysis with one-way ANOVA with Tukey’s multiple comparison test revealed no significant differences.

Superoxide anion levels were measured by HPLC analysis in the aorta (Figure 3A) and the heart (Figure 3B). These tissues all showed a consistent upward trend. These results coupled well with the increases in the marker of lipid peroxidation, MDA-positive proteins in the heart (Figures 3C,D) as well as leukocyte-produced ROS in whole blood, indicating that NOX-2 bearing leukocytes are likely the culprits behind the excessive ROS production (Figures 4A,B). However, whereas oxidative burst showed a maximum for both stimuli, the protein kinase C activating phorbol ester and the Toll-like receptor agonist zymosan A at 4d and 7d of noise exposure (Figure 4A,B), the overall aortic ROS production as measured by DHE staining revealed the most pronounced signal after 28d of noise (Figures 4C,D). According to the cardiac protein expression data, there was overall a trend of higher levels of the vasoconstrictor ET-1 and the oxidative stress markers (NOX-2 and 3-NT) that supports the persistent adverse effects of noise, and even accumulation to some extent) over an exposure duration of 28d (Figure 5). Although this trend was less pronounced for the immunohistochemical data on aortic ET-1, NOX-2 and 3-NT, these vascular data more or less mirrored the blotting data on cardiac ET-1, NOX-2 and 3-NT (Figure 6). Also sNox2-dp serum levels showed an increase by trend in mice exposed to 1, 2 or 4 days of noise (not shown).

### Effects of Long-Term Aircraft Noise on Cerebral Oxidative Stress and Neuroinflammatory Phenotype

**FIGURE 7:**
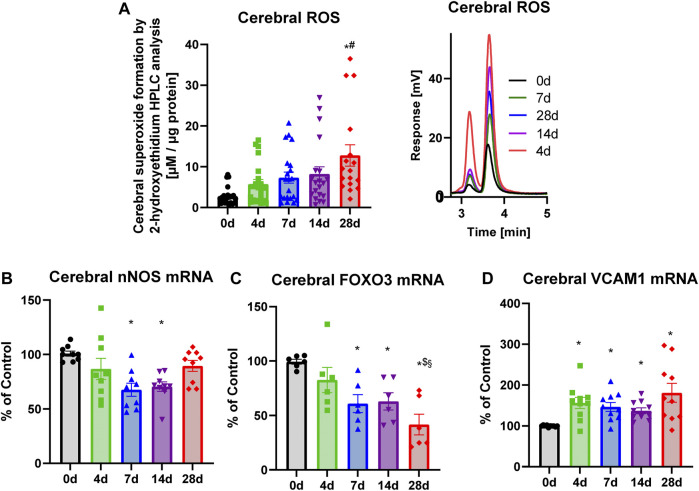
Effects of long-term noise aircraft exposure on noise-induced side effects in the brain. **(A)** Similar to superoxide measurements in aorta and blood, brains of noise-exposed mice displayed a progressively increasing superoxide formation. Representative chromatograms for measurements are alongside the quantifications. **(B)** mRNA isolated from these brains revealed a down-regulated *Nos3.* **(C)** Evidence for a decreased antioxidant transcription factor *Foxo3* and with the consequence of a disruption of the circadian rhythm. **(D)** *Vcam1* was consistently upregulated in all exposure groups. Data points for **(A)** represent individual animals, n = 22–23. Data points from **(B,C,D)** represent pools of tissue from two to three animals. Statistical analysis with one-way ANOVA with Tukey’s multiple comparison test. *p* < 0.05: * vs respective to 0d, # to 4d, $ to 7d, § to 14d and + to 28d.

Noise induced a quite linear increase in cerebral superoxide production over the course of 28d noise exposure as determined by HPLC-based quantification of the superoxide-specific product (Figure 7A). The gene expression of *Nos3* was gradually downregulated until day 7 and 14 of noise exposure but was partially normalized after 28d of noise (Figure 7B). In contrast, *Foxo3* gene expression showed a gradual downregulation over time (Figure 7C). *Vcam1* mRNA expression leveled higher in all exposure groups from the beginning (almost similar to endothelial dysfunction that was also present from the beginning of noise exposure and not changed during prolonged exposure (Figure 7D).

### Effects of Long-Term Aircraft Noise on Hearing Threshold as Assessed by Auditory Brainstem Responses

**FIGURE 8:**
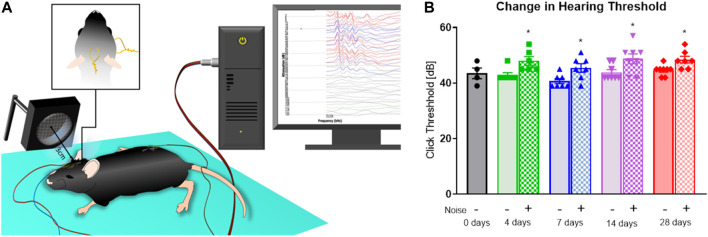
Noise-induced changes in hearing threshold. **(A)** ABR testing was used to evaluate the possibility of noise-induced hearing damage over the course of the noise treatment. The ABR method records the brainstem response to an auditory stimulus in an anesthetized animal using the depicted setup and device. **(B)** Baseline hearing thresholds were consistent, with an apparent rise that was present after only 4 days of noise treatment and appeared to slightly reduce in severity. Data are mean ± SEM. Statistical analysis with one-way ANOVA with Tukey’s multiple comparison test. Points are measurements from individual animals, n = 4–8. *p* < 0.05: * vs respective unexposed group at same time point.

In order to determine whether the hearing of mice was impacted over the long duration of noise exposure, we performed click-evoked ABR testing. ABR testing measures activity in the brain upon hearing an auditory stimulation at various sound pressure levels. The testing was performed both pre- and post-exposure to determine whether noise would cause a raise in the threshold of evoked response. The baseline threshold was consistent throughout the groups, at 43.5 dB at 0d, 42.6 dB at 4d, 40.7 dB at 7d, 43.9 dB at 14d, and 44.6 dB at 28d (Figure 8). In noise-exposed groups, there was a shift in hearing threshold of about 5dB that was apparent by 4 days of noise exposure. The shift did not to progressively worsen throughout the noise exposure, but there was a decrease in the shift over the course of the noise exposure. The changes in threshold were 5.2, 4.8, 4.9, and 3.8 dB, possibly indicating a recovery of this hearing threshold during the treatment course.

## Discussion

With the present studies we assessed our recently developed aircraft noise exposure model over a long-term noise exposure period of 4, 7, 14, and 28d to determine whether the health effects of exposure to aircraft noise observed after 4d of exposure may persist or whether they are subject to adaptation or even tolerance development. To our knowledge, this is the first study investigating the effects of long-term noise stress on the cardiovascular/cerebral system. The results of the present studies clearly indicate that chronic stress leads to manifest arterial hypertension and cerebral effects such as increased ROS production and downregulation of nNOS and FOXO3. There was an acute effect on BP within the first 4d of noise exposure and a further steady increase of more than 20 mmHg during the additional 4 weeks of noise exposure. Increase in BP was associated with a systemic endothelial dysfunction detected as soon as after 4d of noise exposure and a progressive decrease in systemic NO bioavailability and increase in vascular ROS production.

### Consequences of Chronic Noise Exposure on Oxidative Stress and Inflammation

It was an interesting finding that along with the persistent increase in blood pressure, we established steadily increasing ROS formation in the aorta, heart, and brain, which correlated well with the progressive decrease in *Foxo3* mRNA in the brain, a surrogate measure of disruption of the circadian rhythm, which may be in part responsible for the BP effects. In contrast, endothelial dysfunction that was significantly impaired after 4d of noise did not further deteriorate along the 28d exposure period while there was a progressive decline in systemic NO bioavailability and progressive increase in vascular ROS production. Our current concept of cardiovascular effects caused by aircraft noise includes that endothelial dysfunction, which is established already after one night of aircraft noise, is largely triggered by a noise-dependent release of stress hormones, vasoconstrictors such as adrenaline, corticosterone (cortisol in humans) and by an activation of the of the renin-angiotensin-aldosterone system (RAAS) (Munzel et al., 2017) as well as a hypersensitivity of the vasculature to vasoconstrictors (Munzel et al., 2017). In humans the release of the stress hormone cortisol was reported for acute (Gitanjali and Ananth, 2003; Pouryaghoub et al., 2016) and chronic noise exposure (Ising and Ising, 2002; Selander et al., 2009), suggesting that there was no habituation regarding the stress response. These clinical data were also supported by observations in animals that were exposed to acute or chronic noise challenges (Chandralekha et al., 2005; Konkle et al., 2017). Similar data exist for catecholamine release upon acute (Maschke et al., 1993; Schmidt et al., 2013) and chronic noise exposure of humans (Ising et al., 1999; Babisch et al., 2001). A central role of the RAAS in noise-induced cardiovascular damage is supported by our present observation that the ACE inhibitor captopril prevents the noise-dependent blood pressure increase. Activation of the RAAS also provides the link to ROS formation via NOX-2 as AT1-receptor activation produces diacylglycerol, a potent endogenous activator of protein kinase C, which in turn is the major activator of NOX-2 via phosphorylation and translocation of p47phox (reviewed in (Frenis et al., 2021b)). NOX-2 activation was further supported by sNox2-dp serum levels upon noise exposure. Vascular and cerebral superoxide, mainly induced by the phagocytic NADPH oxidase and by an uncoupled eNOS and nNOS stimulates the formation of endothelin-1 and thus reduces the vascular/cerebral NO bioavailability as envisaged by the reduction of inorganic nitrite in the plasma (Kroller-Schon et al., 2018). Chronic stress induces stress hormone release that in the long run may be responsible for the sustained increase of oxidative stress likely via activation of NOX-2 (Kroller-Schon et al., 2018), resulting in increased production of superoxide.

The lack of further deterioration of endothelial function may indicate that there is a mitigating mechanism (“rescue” pathway) that prevents the observed further increases in oxidative stress within the vasculature being translated into a further deterioration of vascular function. We have previously reported that transcription factor Nrf2 may represent an important defense system for preventing the cardiovascular effects noise and that the expression of a down-stream target of Nrf2, heme oxygenase 1, is also induced by noise exposure while concentrations of its antioxidant product bilirubin are diminished (Jimenez et al., 2021). Therefore, it seems likely that an additional antioxidant mechanism may exist that mitigates further exacerbation of a stressed phenotype. Given that the plasma nitrite, aortic NOX-2 expression, and cardiac 3-NT measurements all begin to dip at the 14d time point, it appears that this antioxidant mechanism is becoming effective. Since Nrf2 is known to suppress macrophage inflammatory responses (Kobayashi et al., 2016), a mechanism that has been demonstrated by us to be critical in incurring noise-induced damage (Frenis et al., 2021a), it seems likely that Nrf2-mediated transcription may be protective within this time frame, which however remains a speculation in the context of the present study. This would be also in accordance with the here observed reduced whole blood oxidative burst indicating that activation of inflammatory cells by noise is to some extent reduced between 7 and 14d of noise exposure. The latter concept is also reflected by the maximum of aortic NOX-2 presence (most likely from infiltrated immune cells) at day 7 and decreasing cardiac 3-NT levels (most likely from inducible NOS from infiltrated immune cells) at 14d of continuous noise. We have previously determined that infiltrating leukocytes play a critical role in the effects of noise on the vessels through observations that NOX-2 global knockout mice (Kroller-Schon et al., 2018) and mice with selective ablation of LysM^+^ cells (Frenis et al., 2021a) were protected from the effects of noise as well as from *trans*-endothelial trafficking of monocytes into the aorta.

It remains to be determined why ROS formation in various organs increases further although immune cell activation and infiltrations is prevented likely via endogenous antioxidant and anti-inflammatory defense mechanisms. Thus, it is tempting to speculate that the initial NOX-2-dependent ROS formation stimulates secondary ROS sources such as mitochondria or the xanthine oxidase via an already described redox cross-talk (Daiber, 2010; Schulz et al., 2014; Daiber et al., 2017). Whereas we have evidence for stimulation of mitochondrial ROS formation starting at 4d of noise exposure that is based primarily on mitoSOX fluorescence and an incomplete inhibition of cerebral ROS formation by genetic deletion of Nox2 (Kroller-Schon et al., 2018), a role of xanthine oxidase-dependent ROS formation can be assumed from the beneficial effects of the xanthine oxidase inhibitor allopurinol on cochlear damage and hearing loss in response to 60 h of loud noise exposure with a sound pressure level of 90 dB (Seidman et al., 1993) or 125 dB for 1.8 h (Cassandro et al., 2003).

The results of our present studies indicate that cardiovascular/cerebral side effects in response to chronic aircraft noise occur time-dependently and that noise-induced biochemical changes follow complex kinetics. When immune function was monitored for 1, 7 and 21d of noise exposure (white noise, 85 dB, 2–20 kHz), the authors established a time-dependent modulation of immunosuppression and immunoactivation respectively in a rat model (Van Raaij et al., 1996). Peripheral phagocytic activity was suppressed after 1d of noise, whereas IgM levels in the serum were elevated. Proliferation of lymphocytes in the spleen was suppressed after 7d and higher after 21d of noise, whereas activation of natural killer cells in the spleen was upregulated after 1 and 7d but lower after 21d of noise.

### Cerebral Consequences of Chronic Noise Exposure

Of special importance, the present results add to the emerging evidence of (traffic) noise exposure being directly relevant to brain function and risk of neurodegenerative events such as cognitive decline/impairment and dementia. Although there is a strong body of evidence indicating that cardiovascular risk factors and diseases are related to cognitive decline and dementia risk, thus suggesting that noise exposure may indirectly contribute to these outcomes by modulating vascular neuropathology, the role of noise exposure as a direct influencing factor is less evidenced (Paul et al., 2019).

A number of animal studies, including our own previous research [for review see (Hahad et al., 2021)], demonstrated increased levels of cerebral oxidative stress in response to aircraft noise exposure, constituting a major molecular pathway by which environmental stressors such as air pollution (Hahad et al., 2020) and noise may induce functional and structural deterioration of the brain. Adverse redox signaling of and by microglia (immune cells of the brain) associated with microglial dysregulation constitutes a hallmark of various neurological disease phenotypes leading to neuronal damage/loss and amyloid deposition accompanied by decreased cerebral ^•^NO bioavailability via NOX-2 activation and uncoupling of nNOS and subsequent cerebral vascular endothelial dysfunction (Hahad et al., 2021). Indeed, evidence from animal studies suggests that long-term exposure to higher levels of noise is linked to persistent tau pathology, accelerated overproduction of Aβ, and induction of abnormal auditory input to the brain, all of which associates with aberrant changes in the hippocampus and the cortex (Manikandan et al., 2006; Cui et al., 2009; Cheng et al., 2011; Cui et al., 2012a; Cui et al., 2012b).

Epidemiological evidence from the German Heinz Nixdorf Recall study indicates that traffic-related noise exposure is associated with a lower global cognitive score and a mild cognitive impairment (Tzivian et al., 2016b). Interestingly, these associations were pronounced in former and current smokers (significant interaction), indicating that lifestyle risk factors may potentiate the adverse cerebral effects of noise exposure (Tzivian et al., 2016a). Traffic noise is also shown to be associated with impaired total cognition and the constructional praxis domain (neuropsychological assessment battery), which was robust to adjustment for air pollution (Fuks et al., 2019). Other studies also provided evidence that traffic noise exposure increases the risk of dementia and cognitive impairment (Yu et al., 2020). Most recently, a nationwide study from Denmark including almost 2 million adults aged ≥60 years examined the association between long-term exposure to road traffic and railway noise and risk of incident dementia (Cantuaria et al., 2021). The authors revealed that both road traffic noise and railway noise were associated with increased risk of Alzheimer’s disease, while road traffic, but not railway, noise was also associated with an increased risk of vascular dementia. Impairment of cognition and memory by noise is also well supported by the present data indicating down-regulation of cerebral nNOS by noise and thereby loss of the important neurotransmitter nitric oxide, which is involved in long-term memory and cognitive function (Paul and Ekambaram, 2011). Importantly, although Alzheimer’s disease is probably associated with multiple etiologies and pathophysiologic mechanisms, oxidative stress appears as a major part of the pathophysiologic process (Huang et al., 2016). A major role of oxidative stress in noise-mediated neuronal complications was also supported by increased cerebral ROS formation and downregulation of the protective/antioxidant transcription factor FOXO3.

Taken together, although conflicting results exist concerning the relationship between traffic noise exposure and risk of neurodegenerative events, there is a clear pathomechanistic plausibility to assume substantial cerebral side effects of noise, which is importantly highlighted by our present results showing that noise induced a sustained linear increase in cerebral ROS production and down-regulated Foxo3 gene expression over the total period of exposure.

### No Pronounced Hearing Loss in Response to Aircraft Noise Exposure

An important methodological question concerns the possibility of hearing loss in response to prolonged aircraft noise exposure. To address this issue, we first tested whether chronic aircraft noise exposure with the chosen dB levels may have negative effects of the hearing capacity of our animals. We performed click-evoked ABR-testing prior to and post-noise exposure (Figure 8). The results of these experiments showed a near-immediate increase in click threshold with a possible narrowing in difference in the longer exposures, indicating that our noise exposure protocol does have some small impact on hearing, but that elevation of the hearing threshold from 42 to 48 dB(A) is still far below of the applied mean sound pressure level of 72 dB(A) in our model.

## Conclusion

Our present studies demonstrate distinct changes of the cardiovascular/cerebral system in response to a 4-weeks aircraft noise exposure period. Whereas markers of inflammation reach their maximum levels after 7-14d, superoxide levels seem to gradually increase during the noise period suggesting the involvement of different ROS sources with a dominating NOX-2 contribution during the initial phase and an involvement of mitochondrial ROS and xanthine oxidase during the later phase. In addition, the degree of endothelial dysfunction and the observed increases in blood pressure may rely on different regulatory components such as increased stress hormone release in the early phase and activation of the renin-angiotensin-aldosterone system, increased endothelin-1 expression and an increase of vasoconstrictor sensitivity of the vasculature during the later phase. Especially, the observed and persistent neuronal changes such as cerebral ROS formation, downregulation of nNOS and FOXO3 is worrisome as these adverse processes are also features of neurodegeneration and cognitive decline. We conclude that during a long-term aircraft noise period, that there is no adaptation with respect of the cardiovascular/cerebral side effects or tolerance development further substantiating the potential of noise as a cardiovascular risk factor to induce future cardiometabolic diseases.

## Data Availability

The original contributions presented in the study are included in the article, further inquiries can be directed to the corresponding authors.
